# Supplementary material for: Complex‐centric proteome profiling by SEC‐SWATH‐MS
Source: Mol Syst Biol. 2019 Jan 14;15(1):e8438. doi: 10.15252/msb.20188438 (PMC6346213; doi:10.15252/msb.20188438)
Supplement: Supplementary file 8 — Dataset EV7 [file MSB-15-e8438-s008.zip › feature_plots_string/Q9HAV7.pdf]

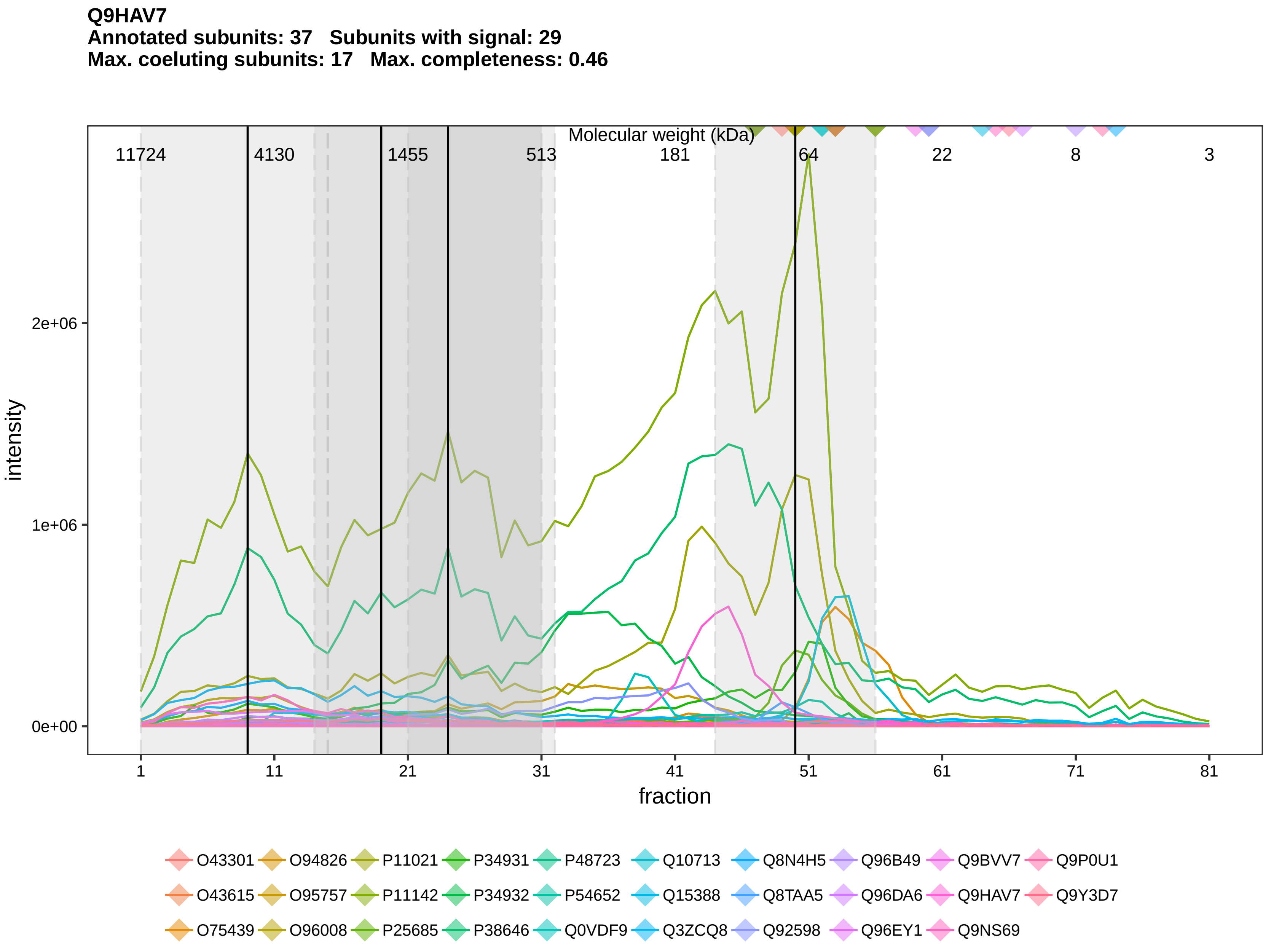

Q9HAV7
Annotated subunits: 37 Subunits with signal: 29
Max. coeluting subunits: 17 Max. completeness: 0.46
Molecular weight (kDa)
11724
4130
1455
513
181
64
22
8
3
intensity
2e+06
1e+06
0e+00
fraction
1
11
21
31
41
51
61
71
81
O43301
O94826
P11021
P34931
P48723
Q10713
Q8N4H5
Q96B49
Q9BVV7
Q9P0U1
O43615
O95757
P11142
P34932
P54652
Q15388
Q8TAA5
Q96DA6
Q9HAV7
Q9Y3D7
O75439
O96008
P25685
P38646
Q0VDF9
Q3ZCQ8
Q92598
Q96EY1
Q9NS69
